# Third Booster Half Dose of ChAdOx1-nCov-19 Is Effective, Safe, and Induces Long-Duration Humoral and Cellular Immune Response to Omicron: 1-Year Follow-Up of Viana Study

**DOI:** 10.3390/vaccines13111113

**Published:** 2025-10-30

**Authors:** Nésio Fernandes de Medeiros-Junior, Maria da Penha Gomes Gouvea, Luiz Antônio Bastos Camacho, Daniel Antunes Maciel Villela, Sheila Maria Barbosa de Lima, Waleska Dias Schwarcz, Adriana Souza Azevedo, Lauro Ferreira Pinto Neto, Carla Magda Allan Santos Domingues, Rosilene Nilo dos Santos Fantoni, Ludimila Forechi, Thaís Ruchdeschel, Laissa Fiorotti Albertino, Matheus Pereira Rosi, Ramon Borge Rizzi, Sara Monteiro Muniz, Hully Cantão dos Santos, Thais Luma de Oliveira Roza, Yasmin Gurtler Pinheiro de Oliveira, Laiza Hombre Dias, Samira Tatiyama Miyamoto, Karina Rosemarie Lallemand Tapia, Danielle Grillo Pacheco Lyra, Jaqueline D’Oliveira Jubini, Ana Paula Neves Burian, Isac Ribeiro Moulaz, Mia Ferreira de Araújo, Luis Fernando Lopez Tort, Any Caroline Alves de Oliveira, Roberta Oliveira Prado, Agnes Antônia Sampaio Pereira, Vitor Hugo Simões Miranda, Elaine Speziali, Christiane Costa-Pereira, Clarice Carvalho Alves, Kétyllen Reis Andrade de Carvalho, Liliane Martins dos Santos, Nani Oliveira-Carvalho, Gabriela de Oliveira, Tâmilla Mayane Alves Fidelis dos Santos, Anna Carolina Cançado Figueiredo, Ismael Artur Costa-Rocha, Ana Carolina Campi-Azevedo, Vanessa Peruhype-Magalhães, Cristiana Couto Garcia, Marilda Mendonça Siqueira, Lis Ribeiro do Valle Antonelli, Jordana Grazziela Alves Coelho-dos-Reis, Andréa Teixeira-Carvalho, José Geraldo Mill, Olindo Assis Martins-Filho, Valéria Valim

**Affiliations:** 1 Secretaria de Estado da Saúde do Espírito Santo (SESA), Vitória 29050-260, Brazil; nesio.junior@gmail.com (N.F.d.M.-J.); laizahombre@gmail.com (L.H.D.); karinalallemand@gmail.com (K.R.L.T.); daniellelyra@saude.es.gov.br (D.G.P.L.); anapaulaburian@gmail.com (A.P.N.B.); 2 Hospital Universitário Cassiano Antônio Moraes (HUCAM-UFES/EBSERH), Programa de Pós-Graduação em Saúde Coletiva (PPGSC), Universidade Federal do Espírito Santo, Av. Mal. Campos, 1355, Santos Dumont, Vitória 29041-295, Brazil; mpgomesgov@gmail.com (M.d.P.G.G.); yasmingurtler@hotmail.com (Y.G.P.d.O.); 3 Escola Nacional de Saúde Pública, Fundação Oswaldo Cruz (FIOCRUZ), Rio de Janeiro 21040-360, Brazil; labcamacho@gmail.com; 4 Programa de Computação Científica (PROCC), Fundação Oswaldo Cruz (FIOCRUZ), Rio de Janeiro 21041-222, Brazil; dvillela@gmail.com; 5 Laboratório de Tecnologia Virológica (LATEV) (Bio-Manguinhos), Fundação Oswaldo Cruz (FIOCRUZ), Rio de Janeiro 21040-360, Brazil; smaria@bio.fiocruz.br; 6 Laboratório de Análise Imunomolecular, Instituto de Tecnologia em Imunobiológicos Bio-Manguinhos (FIOCRUZ), Rio de Janeiro 21040-360, Brazil; waleska.dias@bio.fiocruz.br (W.D.S.); adriana.soares@bio.fiocruz.br (A.S.A.); 7 Escola Superior de Ciências da Santa Casa de Misericórdia de Vitória (EMESCAM), Vitória 29045-402, Brazil; lauropintoneto@gmail.com; 8 Independent Researcher, Brasília 71218-901, Brazil; cmasdomingues@gmail.com; 9 Hospital Universitário Cassiano Antônio Moraes (HUCAM-UFES/EBSERH), Vitória 29041-295, Brazil; rosilenenilo02@gmail.com; 10 Centro de Ciências da Saúde, Medicina, Universidade Federal do Espírito Santo, Vitória 29047-105, Brazil; forechiludmila@gmail.com (L.F.); thais_ruch@hotmail.com (T.R.); laissafiorotti1@gmail.com (L.F.A.); matheus.pereira@gmail.com (M.P.R.); ramonrizzi@gmail.com (R.B.R.); saramonteiro52@gmail.com (S.M.M.); thaisroza.edu@hotmail.com (T.L.d.O.R.); isacmoulaz@gmail.com (I.R.M.); 11 Hospital Universitário Cassiano Antônio de Moraes (HUCAM-UFES/EBSERH), Departamento de Ciências Fisiológicas, Universidade Federal do Espírito Santo, Vitória 29047-105, Brazil; hullycantao@outlook.com (H.C.d.S.); josegmill@gmail.com (J.G.M.); 12 Departamento de Educação Integrada em Saúde, Universidade Federal do Espírito Santo (DEIS/UFES), Vitória 29047-105, Brazil; sa.miyamoto@hotmail.com; 13 Secretaria Municipal de Saúde de Viana, Viana 29130-915, Brazil; jaquelinejubini@hotmail.com; 14 Laboratório de Vírus Respiratórios, Exantemáticos, Enterovírus e Emergências Virais, Instituto Oswaldo Cruz, FIOCRUZ, Rio de Janeiro 21040-900, Brazil; araujo.ferreira.mia@gmail.com (M.F.d.A.); fernandolopeztort@gmail.com (L.F.L.T.); anycaroline.oliveira54@gmail.com (A.C.A.d.O.); cristiana.garcia@fiocruz.br (C.C.G.); mmsiq@ioc.fiocruz.br (M.M.S.); 15 Grupo Integrado de Pesquisas em Biomarcadores, Instituto René Rachou, Fundação Oswaldo Cruz (FIOCRUZ-Minas), Av. Augusto de Lima, 1715, Belo Horizonte 30190-002, Brazil; robertaprado@gmail.com (R.O.P.); agnes.sampaio@hotmail.com (A.A.S.P.); vitor.miranda@fiocruz.br (V.H.S.M.); elaine.faria@fiocruz.br (E.S.); christiane.pereira@fiocruz.br (C.C.-P.); claracarvalves@gmail.com (C.C.A.); ketyllen.andrade@fiocruz.br (K.R.A.d.C.); lilianemartins.bh16@gmail.com (L.M.d.S.); nocarvalho@aluno.fiocruz.br (N.O.-C.); gabrielaoliveira_16@hotmail.com (G.d.O.); tamillamayane@gmail.com (T.M.A.F.d.S.); anna.cancado@fiocruz.br (A.C.C.F.); ismaelarocha@gmail.com (I.A.C.-R.); campiazevedo@gmail.com (A.C.C.-A.); vanessaperuhype@gmail.com (V.P.-M.); andrea.teixeira@fiocruz.br (A.T.-C.); 16 Laboratório de Biologia e Imunologia de Doenças Infecciosas e Parasitárias, Instituto René Rachou, Fundação Oswaldo Cruz (FIOCRUZ-Minas), Belo Horizonte 30190-002, Brazil; lis.antonelli@fiocruz.br; 17 Departamento de Microbiologia, Instituto de Ciências Biológicas, Universidade Federal de Minas Gerais, Av. Antônio Carlos 6627, Belo Horioznte 31270-901, Brazil; reisjordana@gmail.com

**Keywords:** COVID-19, vaccine, fractional dose, clinical trial, ChAdOx1 nCoV-19

## Abstract

**Background**: Dose-sparing approaches can be effective in maintaining immunogenicity and safety while expanding vaccine coverage. We previously demonstrated that a half dose of ChAdOx1 nCoV-19 is as effective and immunogenic for primary vaccination. **Methods**: This non-inferiority, non-randomized controlled trial evaluated the effectiveness, humoral, and cellular immune responses of a third booster dose—comparing half-dose and full-dose regimens—in individuals aged 18–49 years, with a 1-year follow-up. **Results**: A total of 2801 participants were enrolled: 2352 received half doses and 449 received full doses. The incidence rate of COVID-19 was 225.0 per 1000 person-years in the half-dose group and 173.8 in the full-dose group, with no significant difference in effectiveness (β = −0.05; 95% CrI: −0.24 to 0.15). No deaths occurred, and hospitalization rates were similar. In a subsample (n = 558), anti-S IgG levels peaked 28 days post-dose and declined by day 180 after the primary series [175 (121–252) vs. 121 (71–208) GMT, *p* < 0.001], but remained elevated after the booster [192.1 (124–297) vs. 550 (380–797) GMT, *p* < 0.001]. Booster antibody levels were similar between groups [592.4 (318–1140) vs. 550 (380–797) GMT]. The half-dose group showed high titers against Omicron and robust T/B-cell responses (e.g., EMCD4, EMCD8, IFN^+^CD4^+^, CD19^+^TNF^+^). **Conclusions**: Fractional half dose of ChAdOx nCov-19 was effective and non-inferior to a full booster dose. Homologous regimen with 3 half doses or 3 full doses induced a similar increase in antibody titers and robust cellular response. ClinicalTrials.gov (NCT05059106).

## 1. Introduction

On 11 March 2020, the COVID-19 pandemic was declared by the World Health Organization (WHO) [1]. COVID-19 remains one of the greatest global public health challenges, with devastating clinical, social, and economic consequences. By January 2024, more than 774 million cases and over 7 million deaths had been officially reported worldwide [2,3]. This critical situation prompted an unprecedented international effort to develop vaccines capable of reducing adverse clinical outcomes, including mortality [4,5]. It is estimated that the global vaccination campaign prevented nearly 20 million deaths during its first year and significantly limited the spread of the virus [6]. As of January 2024, a total of 13.59 billion COVID-19 vaccine doses had been administered globally [7].

The development of COVID-19 vaccines involved an extraordinary and unprecedented scientific effort, with multiple candidates undergoing clinical trials at record speed [8,9]. However, large-scale production and equitable distribution across populations were essential to achieving global pandemic control [10,11]. Several challenges emerged, including high global demand and limited manufacturing capacity. Scaling up vaccine production was a monumental task [11]. Before this pandemic, contract manufacturing networks were not yet in place for several of the leading vaccine platforms, especially those relying on new technologies such as mRNA [11,12]. Moreover, the volume of vaccines required placed a substantial strain on global supply chains for essential inputs like glass vials, syringes, and stabilizing agents [11,12].

As of May 2023, more than 80% of the population in high-income countries such as the United States, Italy, and Japan had received at least one vaccine dose, and 69–83% had completed the primary vaccination schedule. In contrast, in low-income countries such as Yemen and the Democratic Republic of Congo, only 3–15.54% had received at least one dose, and only 2–13% had completed the full vaccination regimen [13]. While 70% of the global population had received at least one dose, this percentage drops to only 29.9% in low- and middle-income countries [13]. Regarding booster doses, just 32% of the global population had received at least one booster as of that date [7].

Booster doses are essential to restore vaccine effectiveness amid waning immunity and the emergence of new variants. Increasing vaccine uptake in developing countries—by completing a two- or three-dose schedule and ensuring equitable vaccine access—could have contributed significantly to pandemic control [13]. Furthermore, hesitancy toward booster doses, even among fully vaccinated individuals, remains a growing concern [14]. The use of fractional vaccine doses has emerged as a feasible strategy to expand coverage and reduce hesitancy.

Half-dose strategies, which have been used successfully for other infectious diseases, may enhance vaccine coverage in low- and middle-income countries by allowing more individuals to be vaccinated with available supplies [15,16,17]. Additionally, half-dose boosters may address barriers such as low acceptance of boosters (due to prior reactogenicity), costs, and supply constraints [14,17]. Evidence from clinical trials suggests that reduced-dose COVID-19 vaccines may elicit strong immune responses comparable to full doses, with fewer adverse events such as fatigue, fever, and local reactions [16,18,19,20].

The Viana Project (ClinicalTrials.gov: NCT05059106), a non-randomized controlled clinical trial, demonstrated that a half-dose (HD) of ChAdOx1 nCoV-19 was as effective, safe, and immunogenic as the full dose (FD) in 29,598 individuals aged 18–49 years. The published results confirmed the effectiveness of primary vaccination with half-dose ChAdOx1 nCoV-19 in preventing new COVID-19 cases within 90 days of the second dose and in reducing moderate and severe disease during long-term follow-up [21,22].

The present study is an extension of the Viana Project, conducted in 2021 by the Half-Dose ChAdOx Study Group. It focuses on evaluating the humoral and cellular immune responses to SARS-CoV-2 over a one-year period—from primary vaccination with ChAdOx1 nCoV-19 through administration of a third, half-dose booster.

## 2. Population, Materials and Methods

### 2.1. Study Population and Design

This prospective longitudinal study employed a convenience non-probability sampling strategy to evaluate the immunogenicity and effectiveness of a half-dose (0.25 mL) of ChAdOx1 nCoV-19 (ChAd Half Dose) compared to the full dose (ChAd Full Dose) in both the two-dose primary vaccination series and the booster dose among adults residing in Viana, Espírito Santo State, Brazil.

**Figure 1:**
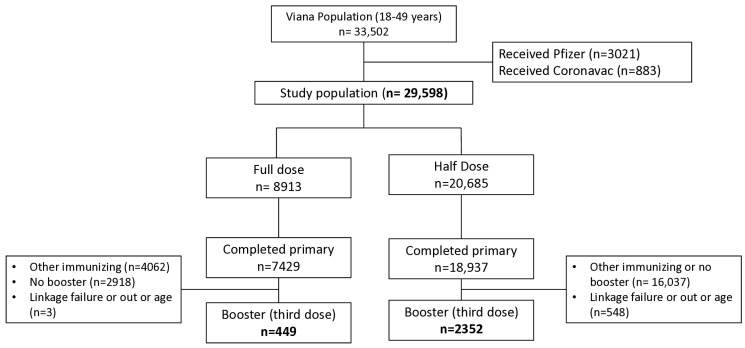
Study flowchart.

Participants included males and females aged 18 to 49 years who were part of the Viana Project and had completed the primary and booster vaccination regimen with ChAdOx1 nCoV-19. Exclusion criteria included receipt of other vaccines, loss to follow-up, or age outside the eligible range (Figure 1). Individuals were referred through the Public Health System’s primary care services, which performed pre-screening to exclude the following: pregnancy; history of severe allergic reaction (anaphylaxis) to any vaccine; vaccination within the previous 14 days; presence of fever or flu-like symptoms; prior COVID-19 vaccination; or recent COVID-19 diagnosis within 28 days before vaccination. The Viana Project protocol and study population have been previously described in detail [21].

For immunogenicity analyses, the ChAd Half Dose group comprised a subgroup of Viana residents, while the ChAd Full Dose group included healthcare workers from the University Hospital of the Federal University of Espírito Santo (HUCAM-UFES/EBSERH) who received the full-dose regimen.

**Figure 2:**
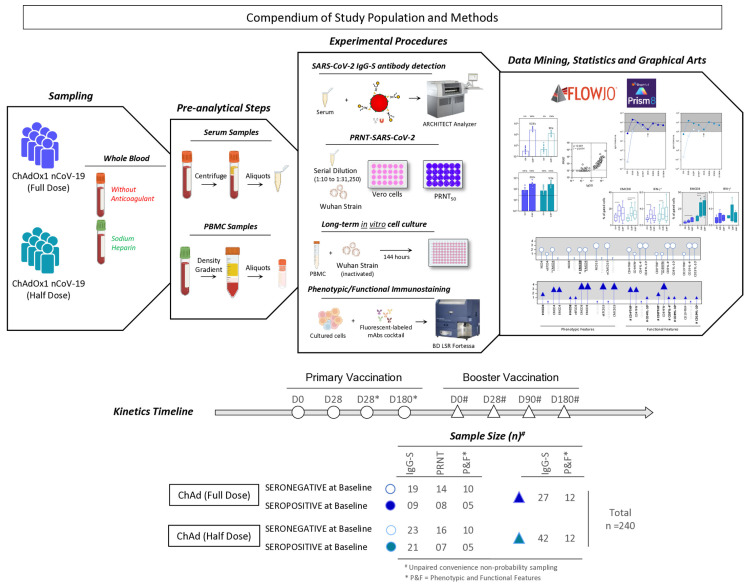
Compendium of study population and methods.

At enrollment, participants were stratified by the baseline IgG anti-S serological status. Figure 2 provides details of groups, sample sizes, study procedures, and analytical approaches.

A comprehensive summary of the study population and methodology is presented in the compendium of study population and methods. This research was designed as a prospective longitudinal investigation, utilizing a convenience non-probability sampling method, to assess the humoral and cellular immune responses following primary vaccination with ChAdOx1 nCoV-19 full dose [ChAd (Full Dose)] and ChAdOx1 nCoV-19 half dose [ChAd (Half Dose)] at consecutive timepoints (D0, D28, D28*, and D180*) up to the administration of a third-booster dose (D0#, D28#, D90#, and D180#). The * and # refer to timepoints related to the second dose and the booster COVID-19 vaccination, respectively. A total of 240 whole blood samples were collected using vacuum systems without anticoagulants to obtain serum samples from each volunteer, and with sodium heparin as an anticoagulant to obtain their peripheral blood mononuclear cell (PBMC) samples. The sample size for each group was determined based on the serological status of each subject at baseline, whether seronegative or seropositive. Serum samples were employed for the detection of SARS-CoV-2 IgG-S antibodies through chemiluminescent microparticle immunoassay and for measuring SARS-CoV-2 neutralizing antibodies via micro plaque reduction neutralization (PRNT) assays. PBMC samples were submitted to long-term *in vitro* cell culture assays followed by phenotypic and functional immunostaining. Data mining and statistics were carried out using FlowJo (V10.6.2) and GraphPad Prism (V9.1.1) software. Distinct approaches for graphical arts were employed, including kinetic profile of reactivity of IgG-S and neutralizing antibody, overall agreement of IgG-S and PRNT titers, analysis and signature profile of phenotypic/functional cellular immunity.

### 2.2. Biological Samples

Blood samples were collected using vacuum tubes: 5 mL without anticoagulant for SARS-CoV-2-specific chemiluminescent microparticle immunoassays and plaque reduction neutralization tests (PRNT), and 20 mL with sodium heparin for phenotypic and functional cellular immune analyses.

A detailed one-year timeline was established for sample collection at the following timepoints: before first dose (D0); 28 days post-first dose (D28); 28 days post-second dose (D28*); 180 days post-first dose (D180*); pre-booster (D0#); and at 28 (D28#), 90 (D90#), and 180 (D180#) days post-booster. The * and # refer to timepoints related to the second dose and the booster COVID-19 vaccination, respectively.

### 2.3. Safety

Safety data regarding vaccine reactogenicity were previously published [21,22]. Hospitalizations and deaths among all residents of Viana were monitored throughout the one-year follow-up.

### 2.4. Effectiveness

Effectiveness endpoints included the incidence rate of confirmed SARS-CoV-2 cases per 1000 person-years within 90 days after the second dose (plus a 14-day window), as identified by RT-PCR in the National Laboratory Management System (GAL) and cases reported in the National Health Surveillance Database (e-SUS VS) [21].

Hospitalizations and deaths were monitored using data from the Espírito Santo State Hospital Bed Management System, the hospital discharge database (AIH), the e-SUS vs. system, the Mortality Information System (SIM), and the Epimed database [21].

### 2.5. Immunogenicity

#### 2.5.1. Detection of SARS-CoV-2 IgG-S Antibodies

IgG antibodies specific to the SARS-CoV-2 spike receptor-binding domain (IgG-S) were quantified using the SARS-CoV-2 IgG II Quant Assay (Abbott Laboratories, Chicago, IL, USA) on the ARCHITECT i1000SR immunoassay analyzer. Results were expressed in binding antibody units per milliliter (BAU/mL), applying the WHO standard conversion factor (1 BAU/mL = 0.142 × AU/mL). Seropositivity was defined as ≥7.1 BAU/mL [23,24].

#### 2.5.2. SARS-CoV-2 Plaque Reduction Neutralization Test (PRNT)

Neutralizing antibodies were measured at the Oswaldo Cruz Institute/FIOCRUZ (Rio de Janeiro, Brazil) in BSL-3 facilities using PRNT with the Wuhan and Omicron SARS-CoV-2 strains. Serial serum dilutions were incubated with viral particles and applied to Vero cell monolayers. Titers were expressed as PRNT50 (≥1:12.6 considered positive) and PRNT90 for Omicron (≥1:10.0 considered positive) [21].

#### 2.5.3. Phenotypic and Functional Memory Cell Assays

SARS-CoV-2-specific T and B-cell responses were evaluated by lymphoproliferation assays performed at the Grupo Integrado de Pesquisas em Biomarcadores, IRR/FIOCRUZ-Minas. PBMCs (2.5 × 10^5^ cells/well) were stimulated with SARS-CoV-2 antigens and analyzed by flow cytometry (BD LSR Fortessa). Memory subsets and cytokine-producing cells were characterized using monoclonal antibody panels from BD Bioscience. FlowJo v10.8 software was used for analysis. T-cell subsets were defined as Naïve, Early Effector, Central Memory, and Effector Memory; B-cell subsets as Naïve, Early Effector, Non-classical Memory, and Classical Memory. Cytokine profiles for TNF-α, IFN-γ, IL-4, and IL-10 were assessed.

### 2.6. Ethical Statements

This study was conducted by the University Hospital of the Federal University of Espírito Santo (HUCAM-UFES/EBSERH) and registered at ClinicalTrials.gov (NCT05059106). It was approved by the National Research Ethics Committee (CONEP, Protocol No. 4.752.775/2021) and the Ethics Review Committee of the Pan American Health Organization (PAHOERC, Protocol No. 0367.02/2021). The use of ChAd Full Dose samples from the HUCAM-UFES/EBSERH biorepository was approved by HUCAM’s Ethics Committee (No. 4.513.439/2021). All participants provided online informed consent. The study adhered to the Declaration of Helsinki, Brazilian national ethical guidelines, and international Good Clinical Practices.

### 2.7. Statistical Analysis

#### 2.7.1. Effectiveness Outcomes

Previously published analyses described the evaluation of primary vaccination effectiveness [21]. For this study, booster dose effectiveness was assessed using a non-inferiority mixed-effects Poisson model, adjusted for age group and sex. The model compared two-dose versus two-dose-plus-booster regimens. Statistical significance was determined using Markov Chain Monte Carlo (MCMC) simulations and evaluated through the 95% credibility interval (CrI) of the group coefficient.

#### 2.7.2. Immunogenicity Outcomes

Statistical analyses were conducted using GraphPad Prism v9.1.1. Non-parametric tests (Mann–Whitney) were applied due to data distribution (Shapiro–Wilk test). A *p*-value ≤ 0.05 was considered significant. Intragroup differences were marked with *, and intergroup comparisons with #.

Correlations between IgG-S and PRNT titers were assessed via the Spearman rank test, and agreement was determined by the Kappa index.

Biomarker signature analyses were conducted in Microsoft Excel 365. Cell marker frequencies were transformed into categorical variables based on global median cut-offs. Lollipop charts highlighted cell features above the median at multiple timepoints (0, 1, 2, 3 or 4) during primary and booster vaccination, with emphasis on consistent changes (gray background).

## 3. Results

### 3.1. Safety Endpoint

During a one-year follow-up, there were 12 hospitalizations of individuals 18 to 49 years old because of COVID-19. Five received primary vaccination with 2 full doses and a booster with BioNTech (BNT) vaccine. One received 2 half-doses and 1 Pfizer booster dose, 2 received only 2 half-doses, 1 only 2 full doses, and 3 received different immunizers in the primary vaccination. No individuals with 3 full doses or 3 half doses were hospitalized.

There were only 2 deaths, both received 2 full doses in primary vaccination and had comorbidities. One had HIV and received a BNT booster dose. Others did not receive any booster dose and had neurological disease. No death was reported in the primary and booster half-dose.

### 3.2. Effectiveness Endpoint

A total of 2801 individuals were included in the study population: 2352 subjects received half doses (ChAd Half Dose) and 449 received full doses (ChAd Full Dose) (Figure 1). The ChAd Full Dose had 206 females of 37.3 ± 8.1 years old, and 189 males of 37.5 ± 8.1 years old; and the Half Dose with 1166 males (32.4 ± 8.8 years) and 1118 males (33.6 ± 8.9 years). The groups were similar in sex and age.

**Figure 3:**
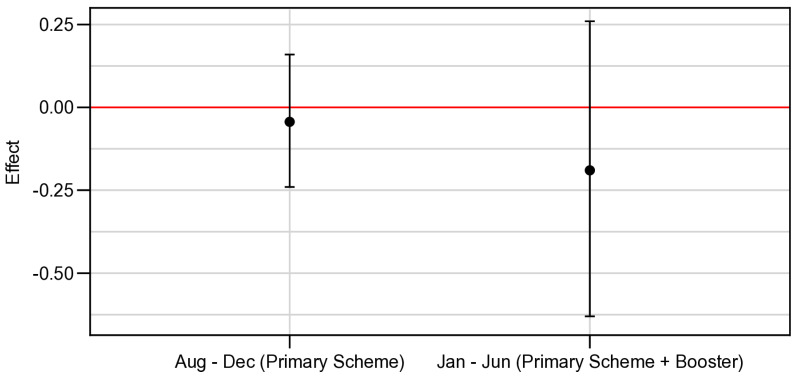
Effectiveness of the primary and booster dose regimen. Effectiveness of the primary and booster with 3 doses of ChAdox1 in the one-year follow-up of the Viana study (incidence/1000 person-year).

The incidence for the ChAd Half Dose group was 225, and for the Full Dose group was 173.8 new cases/1000 persons-year (Table 1). There was no significant difference in the estimated effectiveness comparing groups [ß = −0.05 (95% CrI: −0.24–0.15) for Primary Scheme and ß = −0.20 (95% CrI: −0.63–0.26) for Primary Scheme + Booster] (Figure 3).

During the one-year follow-up, there were twelve hospitalizations of people with 18–49 years, because of SARS-CoV-2: 5 had received full ChAdOx + full ChAdOx + RNAm Pfizer/BioNTech; 2 received half ChAdOx + half ChAdOx + RNAm Pfizer/BioNTech; 2 received 2 half doses of ChAdOx, and 1 received 2 full doses of ChAdOx. The other 3 residents received other platforms in the primary immunization and were excluded.

There were only 2 deaths. One had HIV and received 2 full doses and a booster with RNAm Pfizer/BioNTech. The other had a neurological disease and received 2 full doses of ChAdOx. No death was registered among healthy individuals.

### 3.3. SARS-CoV-2 IgG-S Reactivity and Neutralizing Antibody upon Primary COVID-19 Vaccination

**Figure 4:**
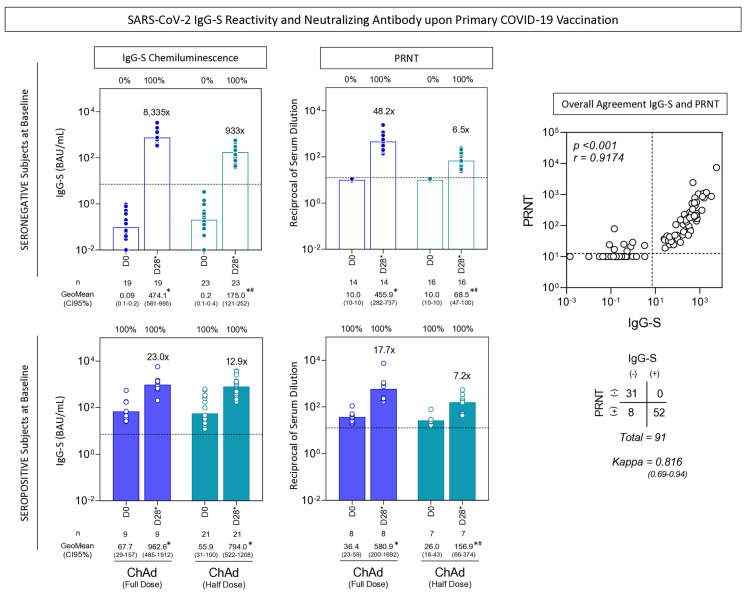
SARS-CoV-2 IgG-S Reactivity and Neutralizing Antibody upon Primary COVID-19 Vaccination. The * and # refer to timepoints related to the second dose and the booster COVID-19 vaccination, respectively.

SARS-CoV-2-specific IgG-S and neutralizing antibody titers were assessed in serum samples collected from individuals, classified as seronegative and seropositive at baseline, upon primary COVID-19 vaccination with ChAdOx1 Full Dose and Half Dose. The results are presented in Figure 4. Data analysis revealed that, in general, both vaccination schemes elicited higher IgG-S and PRNT titers, regardless of the participant’s serological status at baseline, resulting in 100% seroconversion across all groups. Notably, the seronegative subjects displayed a more significant increase in titers at D28*, particularly those who received the ChAd Full Dose (IgG-S—8335× vs. 933×; PRNT—48.2× vs. 6.5×).

Furthermore, we conducted an in-depth evaluation of the concordance between IgG-S and PRNT titers. Our findings demonstrated a strong positive correlation (r = 0.9174, *p* < 0.001), indicating a great overall agreement [Kappa = 0.816 (0.69–0.94)] (Figure 4).

The levels of IgG antibodies to the SARS-CoV-2 spike RBD (IgG-S) and SARS-CoV-2 specific-neutralizing antibody titers were quantified in serum samples from subjects with seronegative (white bars) and seropositive (blue bars) status at baseline at two timepoints: prior the first dose (D0) and 28 days after the second dose (D28*) including subjects receiving ChAdOx1 nCoV-19 full dose [ChAd (Full Dose)] and ChAdOx1 nCoV-19 half dose [ChAd (Half Dose)]. The IgG-S levels and neutralizing antibody titers were determined by the chemiluminescent microparticle immunoassay and micro plaque reduction neutralization test (PRNT), respectively, as described in the Materials and Methods. The IgG-S levels were expressed in BAU/mL, while PRNT titers were expressed as the reciprocal of serum dilution. Data are presented as a scatter plot over bars representing the geometric mean (GeoMean) of IgG-S levels and PRNT titers. The seropositivity was defined considering values above the cut-off edge (dashed line, ≥7.1 BAU/mL for IgG-S levels and ≥1:12.6 for PRNT titers). Comparisons of IgG-S levels and PRNT titers between subgroups (D0 vs. D28*) were carried out by the Mann-Whitney test and highlighted with a * symbol. Significant differences for intergroup (Full Dose vs. Half Dose) comparisons were highlighted by the # symbol. All significant differences were considered at *p* ≤ 0.05. The magnitude of increase for IgG-S and PRNT titers at D28* is provided in the figure. The correlation between the IgG-S and PRNT titers was performed by Spearman’s rank test, and agreement was calculated using the Kappa index to assess the overall agreement between serological tests. The results are presented as a scatter distribution for paired samples, and the scores are provided in the figure.

### 3.4. Kinetics Timeline of IgG-S Reactivity Following Primary and Booster COVID-19 Vaccination

**Figure 5:**
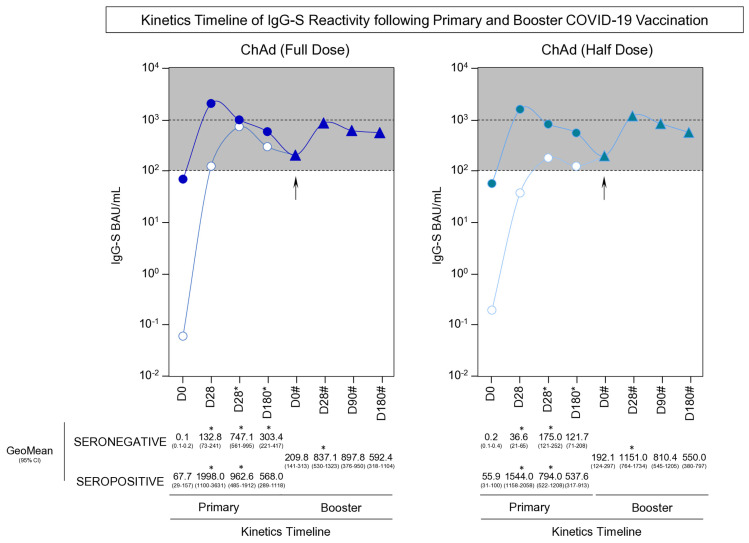
Kinetics Timeline of IgG-S Reactivity following Primary and Booster COVID-19 Vaccination. The levels of IgG antibodies to the SARS-CoV-2 spike RBD (IgG-S) and SARS-CoV-2 specific-neutralizing antibody titers were quantified in serum samples from subjects with seronegative (white symbols) and seropositive (blue symbols) status at baseline. Serum samples were obtained at consecutive timepoints: prior the first dose (D0), 28 days after the first dose (D28), 28 days after the second dose (D28*), 180 days after the first dose (D180*) as well as before (D0#), 28 days (D28#), 90 days (D90#) and 180 days after the booster dose (D180#) including subjects receiving ChAdOx1 nCoV-19 full dose [ChAd (Full Dose)] and ChAdOx1 nCoV-19 half dose [ChAd (Half Dose)]. The * and # refer to timepoints related to the second dose and the booster COVID-19 vaccination, respectively. The timepoints of primary vaccination were represented by circles, while the booster dose timepoints were characterized by triangles. The day of the booster dose is marked by an arrow in the figure. The levels of IgG-S were determined by the chemiluminescent microparticle immunoassay as described in the Materials and Methods. The results are expressed as BAU/mL and presented in line charts representing the geometric mean (GeoMean). The dashed line underscores the IgG-S reactivity ranging from 100 to 1000 BAU/mL. The IgG-S levels were compared with the immediately earlier timepoint. Comparisons were carried out by Mann-Whitney test and highlighted with * symbol. All significant differences were considered at *p* ≤ 0.05.

A comprehensive analysis of the SARS-CoV-2-specific IgG-S reactivity along the kinetics timeline (D0, D28, D28*, D180*, D0#, D28#, D90#, and D180#) focused on participants with seronegative and seropositive status at baseline upon primary COVID-19 vaccination with ChAdOx1 Full Dose and Half Dose is shown in Figure 5.

The results demonstrated that despite the initial serological status and the specific vaccination schemes, our findings reveal a distinctive bimodal trend within all participant subgroups. This pattern is characterized by an initial peak in IgG-S reactivity occurring at D28 or D28*, followed by a decrease in reactivity before the administration of the booster dose (D0#), and a subsequent resurgence in reactivity seen at D28#. Notably, while participants with seronegative status at baseline receiving the ChAd (Full Dose) exhibited an early rise in IgG-S titers exclusively at D28, seronegative subjects receiving the ChAd (Half Dose) displayed a slightly delayed increase at D28* (Figure 5—highlighted in grey rectangles).

Moreover, our data underscores that the ChAd (Half Dose) group exhibited similar IgG-S titers following the booster dose as compared to the ChAd (Full Dose) group (Figure 5).

### 3.5. Neutralizing Antibody Against Wuhan and Omicron SARS-CoV-2 Variants Following Booster COVID-19 Vaccination

**Figure 6:**
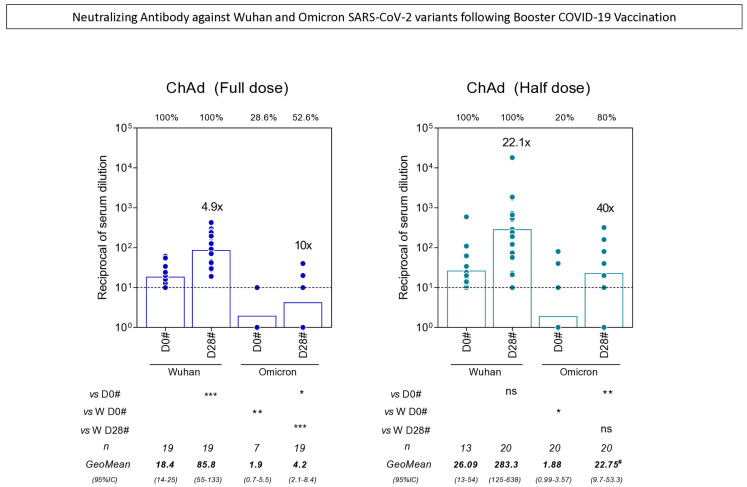
Neutralizing Antibody against Wuhan and Omicron SARS-CoV-2 variants following Booster COVID-19 Vaccination. The levels SARS-CoV-2 Wuhan and Omicron specific-neutralizing antibody titers were quantified in serum samples from subjects with seronegative status at baseline at two timepoints: prior to the booster (D0#) and 28 days after the booster (D28#) including subjects receiving ChAdOx1 nCoV-19 full dose [ChAd (Full Dose)] and ChAdOx1 nCoV-19 half dose [ChAd (Half Dose)]. The # refer to timepoints related to the booster COVID-19 vaccination. The neutralizing antibody titers were determined by microplaque reduction neutralization test (PRNT), as described in the Materials and Methods. The PRNT90 titers were expressed as reciprocal of serum dilution. Data are presented as a scatter plot over bars representing the geometric mean (GeoMean) of PRNT titers. The seropositivity was defined considering values above the cut-off edge (dashed line, ≥1:10 for PRNT90 titers). Comparisons of PRNT titers between subgroups (D0# vs. D28# or Omicron vs. Wuhan) were carried out by paired or unpaired T-test, respectively, and highlighted with * symbol. Differences for intergroup (Full Dose vs. Half Dose) were tested by Mann-Whitney test and were highlighted by # symbol. All significant differences were considered at *p* ≤ 0.05. Seropositivity for each subgroup is presented above the graphs, and fold increase between D0# and D28# is shown above each D28# bar. * *p* < 0.05; ** *p* < 0.01; *** *p* < 0.001.

Upon the emergence of SARS-CoV-2 variants of concern (VOCs), the need to investigate the neutralizing antibodies against these VOCs elicited by different vaccination protocols arose. By the time of the booster dose, the circulating VOC was the Omicron. The analysis of neutralizing antibodies against the Wuhan and Omicron variants following booster COVID-19 vaccination was performed in a subsample of participants with seronegative status at baseline. The results demonstrated that all subjects from the ChAd (Full Dose) and ChAd (Half Dose) groups were seropositive for Wuhan before the booster dose, while the seropositivity for Omicron was under 30% for both groups. The booster with Full Dose enhanced neutralizing antibody levels to both the Wuhan and Omicron variants, whereas the Half Dose booster enhanced antibody titers to Omicron. When the vaccine protocols were compared, it is possible to observe a higher fold increase in both the Wuhan and Omicron variants and higher antibody titers against Omicron in the ChAd (Half Dose) group (Figure 6).

### 3.6. Phenotypic/Functional Features of SARS-CoV-2-Specific T and B-Cells upon COVID-19 Vaccination

**Figure 7:**
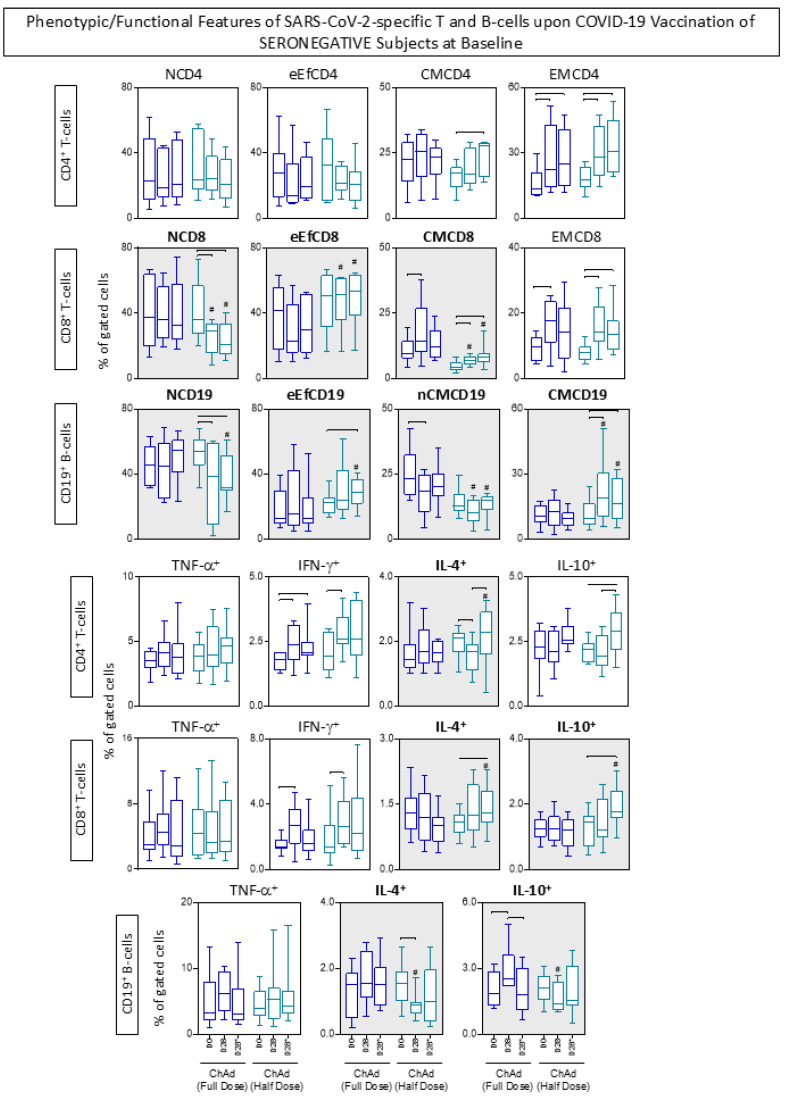
Phenotypic/Functional Features of SARS-CoV-2-specific T and B-cells upon COVID-19 Vaccination of SERONEGATIVE Subjects at Baseline. The SARS-CoV-2-specific T and B-cell features were measured in subjects with seronegative status at baseline at three consecutive timepoints: prior the first dose (D0), 28 days after the first dose (D28) and 28 days after the second dose (D28*) including subjects receiving ChAdOx1 nCoV-19 Full Dose (blue shape) and ChAdOx1 nCoV-19 Half Dose (green shape). The * refer to timepoints related to the second dose of COVID-19 vaccination. The phenotypic and functional features of SARS-CoV-2-specific T and B-cells were evaluated upon *in vitro* SARS-CoV-2 antigen recall of peripheral blood mononuclear cells (PBMC), followed by flow cytometric immunophenotypic staining as described in Materials and Methods. The phenotypic and functional features were expressed as % of gated cells, and the results are displayed in box plot charts. Comparative analysis between subgroups (D0 vs. D28 vs. D28*) was performed by Mann-Whitney test and highlighted with connecting bars. Significant differences for intergroup (Full Dose vs. Half Dose) comparisons were highlighted by # symbol and gray background. In all cases, significant differences were considered at *p* ≤ 0.05.

**Figure 8:**
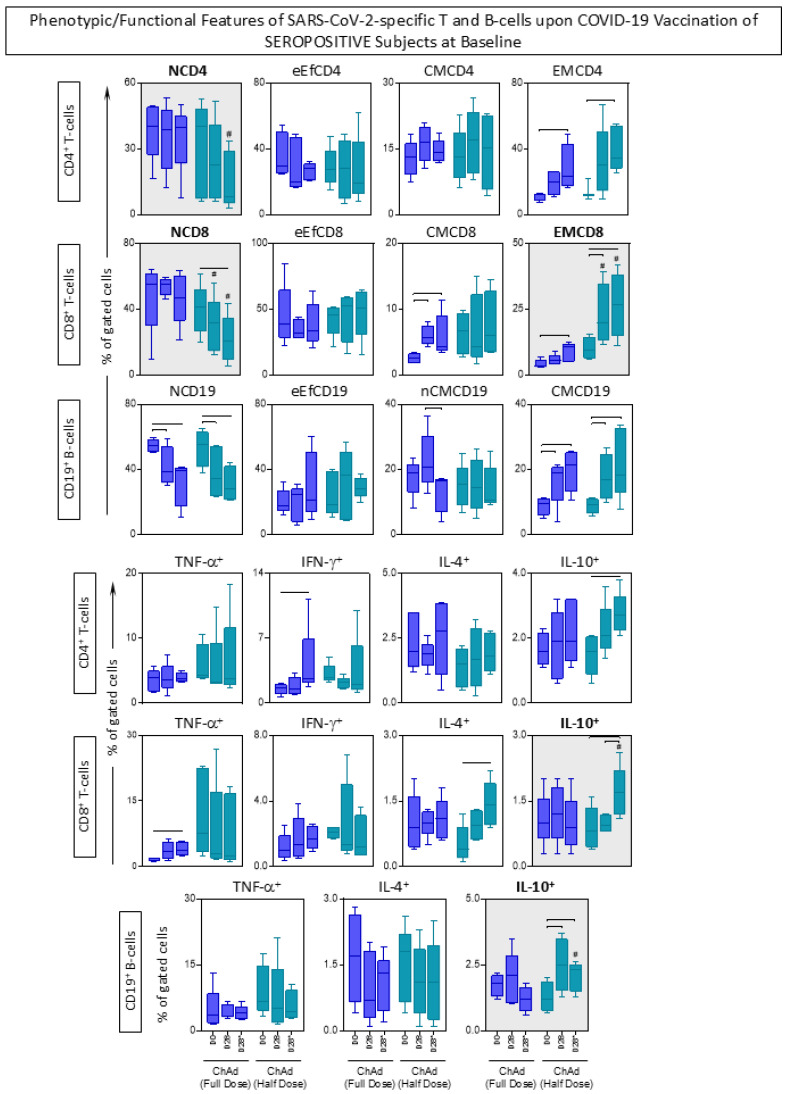
Phenotypic/Functional Features of SARS-CoV-2-specific T and B-cells upon COVID-19 Vaccination of SEROPOSITIVE Subjects at Baseline. The SARS-CoV-2-specific T and B-cell features were measured in subjects with seropositive status at baseline at three consecutive timepoints: prior the first dose (D0), 28 days after the first dose (D28) and 28 days after the second dose (D28*) including subjects receiving ChAdOx1 nCoV-19 Full Dose (blue shape) and ChAdOx1 nCoV-19 Half Dose (green shape). The * refer to timepoints related to the second dose of COVID-19 vaccination. The phenotypic and functional features of SARS-CoV-2-specific T and B-cells were evaluated upon *in vitro* SARS-CoV-2 antigen recall of peripheral blood mononuclear cells (PBMC), followed by flow cytometric immunophenotypic staining as described in Materials and Methods. The phenotypic and functional features were expressed as % of gated cells, and the results are displayed in box plot charts. Comparative analysis between subgroups (D0 vs. D28 vs. D28*) was performed by Mann-Whitney test and highlighted with connecting bars. Significant differences for intergroup (Full Dose vs. Half Dose) comparisons were highlighted by # symbol and gray background. In all cases, significant differences were considered at *p* ≤ 0.05.

The phenotypic and functional features were measured in response to *in vitro* SARS-CoV-2 antigen recall among subjects receiving ChAdOx1 Full and Half dose of COVID-19 vaccination, and the findings are presented in Figure 7 and Figure 8.

Regarding the CD4^+^ T-cells, data analysis revealed that seropositive subjects who received ChAd (Half Dose) exhibited a decrease in NCD4 compared to those who received ChAd (Full Dose). Additionally, there was an increase in effector memory (EMCD4) at day 28 (D28) in all subjects, irrespective of their vaccination scheme or serological status at baseline. However, only in seronegative subjects, we observe early elevated levels of EMCD4 at D28. In line with this approach, analysis of CD8^+^ T-cells features showed that individuals who received ChAd (Half dose) had lower levels of NCD8 throughout the primary vaccination compared to those who received ChAd (Full dose). Furthermore, significant differences in EMCD8 were noted between the vaccination schemes, with increasing levels in the ChAd (Half dose) group at D28 and D28*. Additionally, seronegative subjects receiving the ChAd (Half dose) exhibited increased levels of eEfCD8 and decreased levels of CMCD8 at D28 and D28* compared to the ChAd (Full dose) group. In relation to CD19^+^ B-cells, only seronegative subjects receiving the ChAd (Half dose) showed significant differences between the vaccination schemes, including decreased levels of NCD19 at D28* and nCMCD19 at D28 and D28*, along with increased levels of eEfCD19 at D28* compared to the ChAd (Full dose) group. Both vaccination schemes induced decreased levels of NCD19 and increased levels of CMC19 in seropositive subjects (Figure 7 and Figure 8).

In relation to the functional features, the analysis indicated that differences mostly appeared after the second dose (D28*) in individuals receiving the half dose. Across the groups, there was an equivalent high level of IL-10^+^CD8^+^ at D28*. Seropositive subjects vaccinated with the ChAdOx1 Half Dose displayed increased levels of IL-10^+^CD19^+^ at D28* compared to those vaccinated with the ChAdOx1 Full Dose. Furthermore, the Half Dose led to increased levels of IL-4^+^ T-cell subsets at D28* and a decrease of IL-4^+^ and IL-10^+^ B-cells at D28 (Figure 7 and Figure 8).

Notably, seronegative subjects at baseline exhibited substantial levels of EMCD8 and increased production of IFN-γ by T-cell subsets after 28 days of both full and half-dose vaccinations.

### 3.7. Kinetics Timeline of Phenotypic and Functional Features of SARS-CoV-2-Specific T and B-Cells Following Primary and Booster COVID-19 Vaccination

**Figure 9:**
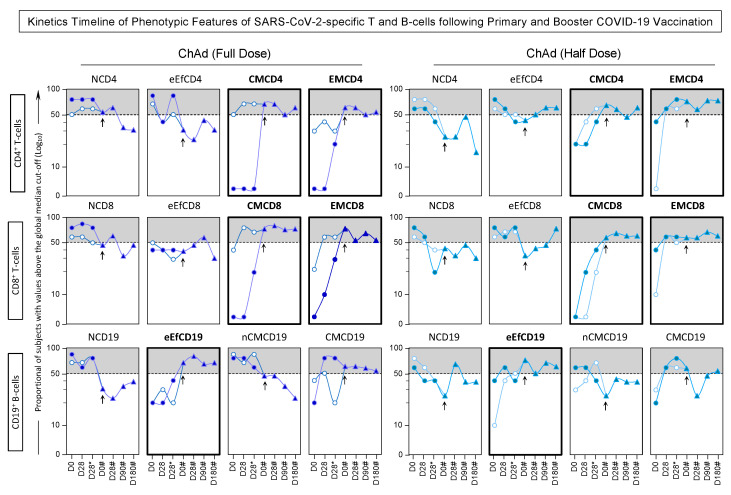
Kinetics Timeline of Phenotypic Features of SARS-CoV-2-specific T and B-cells following Primary and Booster COVID-19 Vaccination. The SARS-CoV-2-specific T and B-cell features were measured in subjects with seronegative (white symbols) and seropositive status (blue symbols) at baseline. Whole blood samples were obtained at consecutive timepoints: prior the first dose (D0), 28 days after the first dose (D28), 28 days after the second dose (D28*), 180 days after the first dose (D180*) as well as before (D0#), 28 days (D28#), 90 days (D90#), and 180 days after the booster dose (D180#) including subjects receiving ChAdOx1 nCoV-19 full dose [ChAd (Full Dose)] and ChAdOx1 nCoV-19 half dose [ChAd (Half Dose)]. The * and # refer to timepoints related to the second dose and the booster COVID-19 vaccination, respectively. The timepoints of primary vaccination were represented by circles, while the booster dose timepoints were characterized by triangles. The day of the booster dose is marked by an arrow in the figure. The phenotypic features of SARS-CoV-2-specific T and B-cells were evaluated upon *in vitro* SARS-CoV-2 antigen recall of peripheral blood mononuclear cells (PBMC), followed by flow cytometric immunophenotypic staining as described in Materials and Methods. The results are expressed as the proportion of subjects with values above the global median cut-off. Data analysis was carried out considering the 50th percentile as the reference to identify the set of features with high proportion of subjects with levels above the global median cut-off along the kinetics timeline. Those cell phenotypes with proportion of subjects with levels above the global median cut-off were highlighted with gray background, and those that maintain levels above the 50th percentile after booster dose (D0#) are underscored in bold frames.

**Figure 10:**
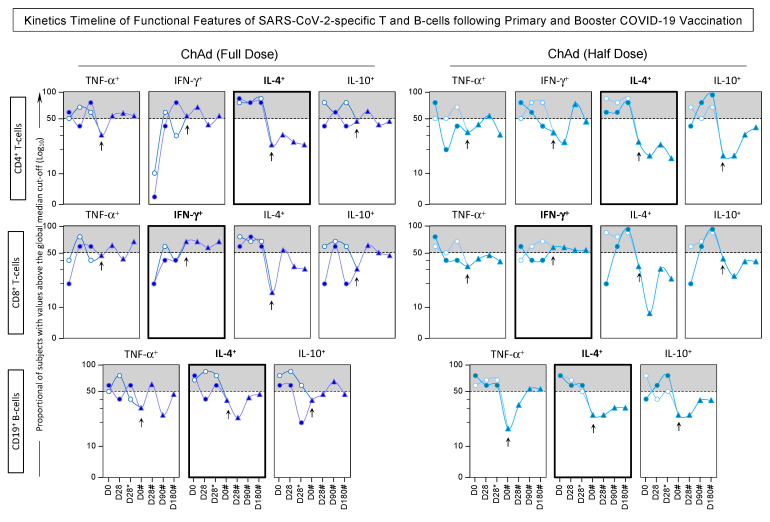
Kinetics Timeline of Functional Features of SARS-CoV-2-specific T and B-cell following Primary and Booster COVID-19 Vaccination. The SARS-CoV-2-specific T and B-cell features were measured in subjects with seronegative (white symbols) and seropositive status (blue symbols) at baseline. Whole blood samples were obtained at consecutive timepoints: prior the first dose (D0), 28 days after the first dose (D28), 28 days after the second dose (D28*), 180 days after the first dose (D180*) as well as before (D0#), 28 days (D28#), 90 days (D90#), and 180 days after the booster dose (D180#) including subjects receiving ChAdOx1 nCoV-19 full dose [ChAd (Full Dose)] and ChAdOx1 nCoV-19 half dose [ChAd (Half Dose)]. The * and # refer to timepoints related to the second dose and the booster COVID-19 vaccination, respectively. The timepoints of primary vaccination were represented by circles, while the booster dose timepoints were characterized by triangles. The day of the booster dose is marked by an arrow in the figure. The phenotypic features of SARS-CoV-2-specific T and B-cells were evaluated upon *in vitro* SARS-CoV-2 antigen recall of peripheral blood mononuclear cells (PBMC), followed by flow cytometric immunophenotypic staining as described in Materials and Methods. The results are expressed as the proportion of subjects with values above the global median cut-off. Data analysis was carried out considering the 50th percentile as the reference to identify the set of features with a high proportion of subjects with levels above the global median cut-off along the kinetic timeline. Those functional features with proportion of subjects with levels above the global median cut-off were highlighted with gray background, and those that maintain levels above the 50th percentile after booster dose (D0#) are underscored in bold frames.

The temporal evolution, regarding the kinetics timeline, of phenotypic and functional features within SARS-CoV-2-specific T and B-cells following primary and booster COVID-19 vaccination was expressed by quantifying the proportion of subjects with values above the global median cut-off for each attribute, and the results are shown in Figure 9 and Figure 10.

Data analysis demonstrates that both the ChAd Full Dose and Half Dose vaccination schemes induced a dynamic pattern of phenotypic and functional features throughout the timeline. Notably, both vaccination schemes consistently elicit raised levels of memory-associated phenotypic attributes upon the administration of the booster dose. These attributes involve CMCD4, EMCD4, CMCD8, EMCD8, and eEfCD19. Simultaneously, there is a decrease in the production of IL-4 by CD4^+^ and CD19^+^ cells and an increase in the production of IFN-γ by CD8^+^ T-cells (Figure 9 and Figure 10).

**Figure 11:**
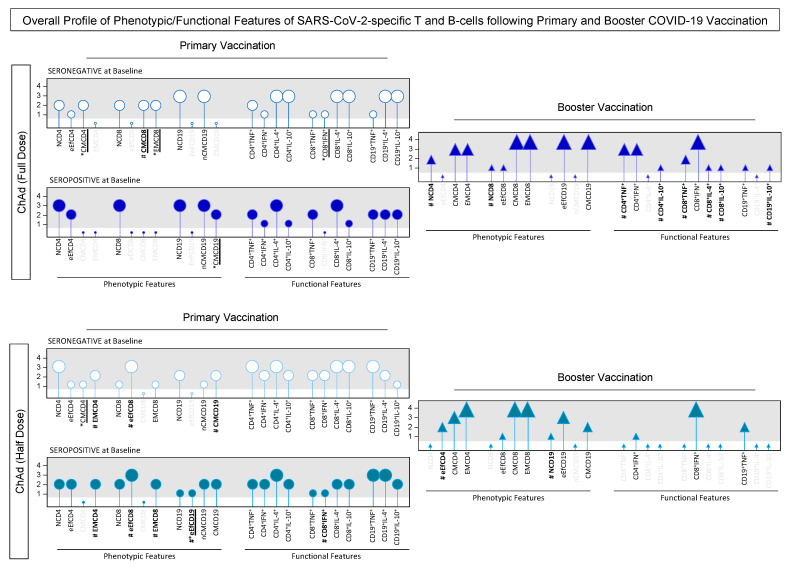
Overall Profile of Phenotypic/Functional Features of SARS-CoV-2-specific T and B-cells following Primary and Booster COVID-19 Vaccination. The SARS-CoV-2-specific T and B-cell features were measured in subjects with seronegative (white symbols) and seropositive status (blue symbols) at baseline. Whole blood samples were obtained at consecutive timepoints: prior the first dose (D0), 28 days after the first dose (D28), 28 days after the second dose (D28*) (Primary Vaccination) as well as before (D0#), 28 days (D28#), 90 days (D90#), and 180 days after the third dose (D180#) (Booster Vaccination), including subjects with seronegative and seropositive results at baseline, receiving ChAdOx1 nCoV-19 full dose [ChAd (Full Dose)] and ChAdOx1 nCoV-19 half dose [ChAd (Half Dose)]. The primary vaccination was represented by circles, while the booster dose was characterized by triangles. The phenotypic and functional features of SARS-CoV-2-specific T and B-cells were evaluated upon *in vitro* SARS-CoV-2 antigen recall of peripheral blood mononuclear cells (PBMC), followed by flow cytometric immunophenotypic staining as described in Materials and Methods. Data are shown in lollipop charts representing the number of timepoints (0, 1, 2, 3 or 4) that the phenotypic/functional features displayed a higher proportion of subjects above 50% considering the global median cut-off along the kinetics timeline at primary or booster vaccination. The gray background highlighted the set of phenotypic/functional features with more than 1 timepoint, along the kinetics timeline, with a higher proportion of subjects above the global median cut-off. Differences for intragroups (SERONEGATIVE vs. SEROPOSITIVE at baseline) are highlighted by an underline format and an asterisk symbol. Differences for intergroup (ChAdOx1 nCoV-19 Full Dose vs. ChAdOx1 nCoV-19 Half Dose) comparisons were highlighted by bold format and # symbol. Parameters with gray writing represents those features with no subjects with levels above the global median cut-off along the kinetics timeline.

A complementary analysis was conducted to assess the comprehensive profile of phenotypic and functional features, considering the number of changes observed over the kinetics timeline during primary and booster vaccination (Figure 11). In general, differences in memory-cell phenotypic features were predominantly concentrated during primary vaccination. Specifically, among seronegative subjects at baseline who received the ChAd (Full Dose) vaccination, elevated levels of CMCD4 and EMCD8 were observed in comparison to seropositive subjects. In addition, CMCD8 also displayed increased levels in contrast to seronegative individuals who received the Half Dose scheme. Within the ChAd (Half Dose) group, seronegative subjects at baseline displayed increased CMCD4 levels as compared to seropositive subjects. Furthermore, EMCD4, eEfCD8, and CMCD19 exhibited differences over the kinetics timeline among seronegative subjects who received the Half Dose scheme when compared to the subjects receiving the Full Dose scheme. Conversely, seropositive individuals at baseline who received the Half Dose vaccination demonstrated increased levels of EMCD4, eEFCD8, EMCD8, and eEFCD19 in comparison to the Full Dose scheme. Contrarywise, only CD8^+^IFN^+^ levels exhibited alterations over the kinetics timeline regarding the primary vaccination (Figure 11).

While differences during primary vaccination predominantly concerned memory-cell phenotypic features, the booster vaccination induced more differences in functional features. Notably, the ChAd (Full Dose) group displayed increased levels of CD4^+^TNF^+^, CD4^+^IL-10^+^, CD8^+^TNF^+^, CD8^+^IL-4^+^, CD8^+^IL-10^+^, and CD19^+^IL-10^+^. In contrast, the ChAd (Half Dose) exhibited increased levels of eEfCD4 and NCD19 (Figure 11).

Remarkably, several crucial features within the vaccination context exhibited enhancements in both schemes, including EMCD4, EMCD8, production of IFN-γ by CD4^+^ and CD8^+^ T-cells, and TNF by CD19^+^ cells.

## 4. Discussion

Dose-sparing strategies for COVID-19 vaccination have been explored to optimize vaccine distribution and coverage, especially in the context of limited supply. Several approaches have been investigated and supported by clinical evidence, including fractional doses, intradermal administration, extending dosing intervals, and single-dose strategies. These strategies are supported by clinical trials and modeling studies, which indicate that dose-sparing approaches can be effective in maintaining immunogenicity and safety while expanding vaccine coverage. Implementing these strategies can be particularly beneficial in resource-constrained settings or during periods of vaccine scarcity [21,25,26,27,28,29].

Our group previously demonstrated that primary vaccination with a half dose of ChAdOx1 nCoV-19 vaccine is non-inferior to full dose regarding effectiveness, safety, and humoral immunogenicity in 90 days of follow-up [21,22]. In the present study, we have demonstrated non-inferiority of the booster using the Half Dose of ChAdOx1 nCoV-19 in a one-year follow-up to protect against Omicron variants. Additionally, we have shown that the Half Dose regimen induced a great cellular response, with involvement of effector memory CD4^+^ and CD8^+^ T-cells and IFN-γ-producing CD4^+^ and CD8^+^ T-cells.

Reducing the dose of the vaccine while maintaining immunogenicity and safety is a key strategy. Studies have shown that fractional doses of mRNA COVID-19 vaccines (e.g., BNT162b2 and mRNA-1273) and adenovirus vector vaccines (e.g., ChAdOx1 nCoV-19) can elicit comparable immune responses to full doses. A systematic review and meta-analysis demonstrated that fractional doses of mRNA and protein subunit vaccines could induce sufficient neutralizing antibodies and T-cell responses, providing reasonable protection against both ancestral and variant strains of SARS-CoV-2. Additionally, a study on the ChAdOx1 nCoV-19 vaccine showed that a half-dose booster after two doses of an inactivated vaccine was non-inferior in terms of immunogenicity and had a lower rate of systemic reactogenicity compared to the standard dose [21,25,26].

We found lower reactogenicity in the half dose compared to the full dose [21]. Now, in a one-year follow-up, there was no death in vaccinated healthy individuals, confirming the safety of the platform and effectiveness of the half dose.

In COVID-19 vaccination strategies, heterologous (or sequential) vaccination is commonly adopted after the primary immunization series, meaning that a different type of vaccine—typically one that is not adenoviral—is administered as the booster. The decision to use ChAdOx1 nCoV-19 as the booster dose in our study was based on several key factors. First, the study was conducted in a context where ChAdOx1 nCoV-19 was the vaccine platform available through the public health system at the time of booster administration. Logistical feasibility and real-world applicability were important considerations, especially in regions where mRNA vaccines were not yet broadly accessible or were being prioritized for specific high-risk groups. Second, although heterologous booster regimens have demonstrated immunological advantages in some studies, homologous boosting with ChAdOx1 nCoV-19 has also been shown to elicit significant increases in neutralizing antibody titers and T-cell responses after a prolonged interval from the primary series [4]. This provided an opportunity to evaluate the immunogenicity and safety of a homologous regimen under real-world conditions. Finally, while adenoviral vector vaccines have been associated with rare adverse events such as vaccine-induced immune thrombotic thrombocytopenia (VITT), these occurrences are extremely uncommon. Surveillance data and clinical studies have shown that the overall safety profile of ChAdOx1 nCoV-19 remains acceptable, particularly when used in adult populations without known contraindications [30,31]. In our study, no serious adverse events were observed following the booster dose, reinforcing the safety of this approach. In summary, the choice of ChAdOx1 nCoV-19 for the booster dose reflected both the practical realities of vaccine deployment in our setting and the scientific rationale for evaluating homologous vector-based boosting strategies.

It is well known that neutralizing antibodies, as well as IgG-S titers, displayed a pivotal role in the COVID-19 vaccination scenario relating to the durability of the vaccine response and the evaluation of the necessity for booster doses [32]. In this line, one key result found in this study was that both humoral aspects evaluated, antibody neutralization and IgG-S titers, following the booster dose, were robust across the Half- and Full-dose groups. Specifically, our data showed that antibody response peaked 28 days after vaccination, which aligns with the growing body of evidence regarding the kinetics observed in other COVID-19 vaccine studies [14,33,34]. Of note, an outstanding peak of antibody neutralization titer was observed after the booster in the Half Dose regimen, contrasting with the Full Dose booster. Similar to other dose-sparing investigations, a reduction in antibody response was noticed after 180 days post-primary vaccination, regardless of the status at baseline, highlighting the importance of booster doses to guarantee the maintenance of correlates of protection [4,33]. This pattern of waning humoral immunity followed by sustained protection post-booster has been observed in other COVID-19 vaccines, including both adenovirus-based and mRNA-based platforms [35]. However, the retention of elevated broad-spectrum IgG-S and neutralizing antibody titers post-booster suggests long-lasting protection even at the Half-Dose regimen [36,37].

From a cellular immunogenicity perspective, the present study revealed a clear shift of naive phenotypes to a robust and functional memory response across the Half- and Full-dose groups. The booster dose enhanced important attributes associated with memory response, such as central and effector memory of CD4^+^ (CMCD4 and EMCD4) and CD8^+^ T-cell phenotypes (CMCD8 and EMCD8), along with increased production of IFN-γ and IL-4 by T-cell subsets. These findings are critical as they indicate not only the generation of long-lasting memory responses but also the functional activity of these cells in mounting an effective immune response against a future infection with SARS-CoV-2. The observed T-cell responses are consistent with other studies that have shown the robust importance of cellular immune response in the COVID-19 context, particularly the involvement of IFN-γ-producing T-cells [38,39,40]. Additional reports have also associated the response and protection displayed by T-cells, acquired through vaccination or infection, with being less vulnerable to genetic variations and virus mutations, as well as to rapid recall responses that can limit viral replication and dissemination in the host, thereby preventing severe disease [41,42,43]. Taking this together, the comparable cellular responses between the Half-dose and Full-dose groups suggest that fractional doses can still induce a robust, long-lasting T-cell-mediated immune response.

Khoury and colleagues [44] pointed out the association of the antibody response with cellular immune compartments, calling attention to studies that address responses such as T-cells and B-cell responses as additional potential correlates of protection after vaccination regimens. Our data demonstrated the presence of effector and memory phenotypes of B-cells all along the kinetics timeline. Booster vaccination resulted in a large increase in Memory B-cell responses in adults [45]. Questions remain regarding T-cell responses following vaccination of either uninfected or previously infected individuals. First, it will be important to understand whether CD4^+^ or CD8^+^ T-cells contribute to the differences observed between vaccine groups. Furthermore, determining the relationship between cTfh responses and neutralizing titers after vaccination of infected subjects will give greater insight into the requirement for T-cell help during recall of memory B-cell responses.

This study is not without its limitations. One limitation of this study is the potential underreporting of post-vaccination SARS-CoV-2 infections, particularly in cases that were mild or asymptomatic. As prior immunity from vaccination or natural infection increases, individuals may experience fewer or less intense symptoms and consequently may not seek medical evaluation or diagnostic testing [30]. This behavior could lead to an underestimation of the true incidence of post-vaccination infections. However, it is important to note that this potential reporting bias likely occurred similarly across both study arms, given the comparable follow-up procedures and communication protocols applied to all participants. Therefore, while the absolute infection rates may be underestimated, the internal validity of the comparative analysis remains preserved. Furthermore, our study’s primary outcomes focused on immunogenicity and safety, which are less susceptible to reporting bias and strengthen the reliability of the findings.

Although parenteral vaccines, such as ChAdOx1 nCoV-19, have proven effective in preventing severe COVID-19 and death, their limited ability to induce mucosal immunity may help explain the occurrence of breakthrough infections. SARS-CoV-2 infects primarily through the respiratory mucosa, and recent studies have shown that mucosal secretory IgA (SIgA), rather than systemic IgG, plays a central role in preventing viral entry and transmission. Bladh et al. (2023) demonstrated that even after a fourth dose of an mRNA vaccine, mucosal IgA responses were poorly boosted, with no correlation between serum IgG and nasal IgA levels [46]. These findings underscore the limitations of systemic immunization in eliciting effective mucosal immune responses and highlight the need for complementary vaccine strategies capable of inducing robust local immunity at the site of viral entry. Nonetheless, the long-lasting systemic humoral and cellular responses observed in our study support the continued use of booster doses to reduce the risk of severe disease, particularly in high-risk populations.

Because of ethical reasons and a short window of opportunity to study fractional doses during the pandemic, it was not possible to implement a randomized clinical trial. The participant decided to be included in the half or full ChAdOx1 nCoV-19, and it could reflect different behavior that could influence different exposure risks, such as wearing a mask and avoiding environments with a large number of people. Different risk behaviors could interfere with the effectiveness outcome. However, it is unlikely that they could interfere with immunogenicity and safety outcomes. It is very interesting to report that the main motivation for participating in the study in the half-dose arm was vaccine hesitancy. Many people are afraid of receiving vaccines, but they know the need for this strategy to control pandemics and infections. Therefore, they opt for less immunogenic platforms, such as inactivated viruses, or schemes with a lower number of doses. Considering that vaccine hesitancy is a major challenge for expanding vaccination coverage, fractionated doses could also be an option to increase vaccine adherence [47].

Unfortunately, after more than three billion doses distributed and millions of lives saved, the ChAdOx1 nCoV-19 vaccine has been withdrawn because of safety concerns. However, efforts are ongoing to try to fix this problem, and other vector viral vaccines, like the Ebola vaccine, are available. Anyhow, fractional low-dose ChAdOx1 nCoV-19 booster should be considered, especially in resource-constrained settings [26], to prevent new SARS-CoV-2 outbreaks or to achieve full world coverage.

As vaccine access in low-income countries remains an extraordinary challenge, studies about fractional doses are definitely important. Fractional dose yellow fever vaccination has been considered as a strategy to address vaccine shortages [48]. As the global vaccine supply eventually can’t handle the multiplicity of outbreaks, as occurred with mpox recently, the immunogenicity of fractional doses must be known.

Our results diversify the evidence base from which policymakers and immunization advisory committees can draw up to make flexible decisions regarding boosting schedules specific to various primary schedules. Fractional dosing may improve COVID-19 booster acceptability and uptake and will reduce the per-dose cost of COVID-19 booster programs, which is particularly important in low- and middle-income countries.

## 5. Conclusions

In conclusion, a fractional half dose of ChAdOx nCov-19 was effective and non-inferior to a full booster dose. Homologous regimen with 3 half doses or 3 full doses induced a similar increase in antibody titers and robust cellular response.

## Figures and Tables

**Table 1:** Effectiveness of the booster dose with half and full doses of ChAdox1 following the Viana study (incidence/1000 person-years) after 180 days follow-up after booster dose vaccination.

		ChAdOx1 Full Dose		ChAdOx1 Half Dose	
Age (years)	Sex	New cases/total person-year	Rate (cases/1000 person-year)	New cases/total person-year	Rate (cases/1000 person-year)
18–29	F	3/4261	257.0	35/63,530	201.1
18–29	M	0/2680	0.0	17/58,208	106.6
30–39	F	8/7244	403.1	35/57,585	221.8
30–39	M	4/6701	217.9	27/49,872	197.6
40–49	F	7/9425	271.1	26/42,877	221.3
40–49	M	2/8621	84.7	13/49,274	96.3
All		24/38,932	225	153/321,346	173.8
Effect	beta	−0.05 (95% CrI: −0.24–0.15)			

Mixed-effects Poisson model. Model-based analysis adjusted for age, sex, time of protection (D3 + 14). New Notified cases registered at e-SUS vs. databank, from January to June. There was no difference between groups.

## Data Availability

The datasets generated and/or analyzed during the current study are available from the corresponding author upon request.

## Abbreviations

AIH	Hospital Discharge Database (Autorização de Internação Hospitalar)
BAU/mL	Binding Antibody Units per Milliliter
BNT162b2	Pfizer-BioNTech COVID-19 vaccine
BNT	BioNTech
BSL-3	Biosafety Level 3
CMCD4	Central Memory CD4^+^ T-cells
CMCD8	Central Memory CD8^+^ T-cells
CONEP	National Research Ethics Committee (Comissão Nacional de Ética em Pesquisa)
COVID-19	Coronavirus Disease 2019
ChAd Full Dose	Full Dose of ChAdOx1 nCoV-19
ChAd Half Dose	Half Dose of ChAdOx1 nCoV-19
ChAd	ChAdOx1 nCoV-19 Vaccine
CrI	Credibility Interval
DECF/UFES	Departamento de Ciências Fisiológicas, UFES
DEIS/UFES	Departamento de Educação Integrada em Saúde, UFES
EBSERH	Empresa Brasileira de Serviços Hospitalares
EMCD4	Effector Memory CD4^+^ T-cells
EMCD8	Effector Memory CD8^+^ T-cells
EMESCAM	Escola Superior de Ciências da Santa Casa de Misericórdia de Vitória
e-SUS VS	National Health Surveillance Database
eEfCD19	Early Effector CD19^+^ B-cells
eEfCD4	Early Effector CD4^+^ T-cells
eEfCD8	Early Effector CD8^+^ T-cells
FD	Full Dose
FIOCRUZ-Minas	Fundação Oswaldo Cruz, Instituto René Rachou, Belo Horizonte, MG, Brazil
FIOCRUZ	Oswaldo Cruz Foundation (Fundação Oswaldo Cruz)
GAL	National Laboratory Management System
HD	Half Dose
HIV	Human Immunodeficiency Virus
HUCAM-UFES	University Hospital of the Federal University of Espírito Santo
IFN-γ	Interferon Gamma
IL-10	Interleukin 10
IL-4	Interleukin 4
IRR	René Rachou Institute (Instituto René Rachou)
IgG-S	Immunoglobulin G-Specific Antibodies to Spike protein
MCMC	Markov Chain Monte Carlo
NCD19	Naïve CD19^+^ B-cells
NCD4	Naïve CD4^+^ T-cells
NCD8	Naïve CD8^+^ T-cells
NCMCD19	Non-classical Memory CD19^+^ B-cells
PAHOERC	Pan American Health Organization Ethics Review Committee
PBMC	Peripheral Blood Mononuclear Cell
PRNT	Plaque Reduction Neutralization Test
PRNT50	Neutralizing Antibody Titers (50%)
PRNT90	Neutralizing Antibody Titers (90%)
PROCC	Programa de Computação Científica, Fundação Oswaldo Cruz
RNAm	mRNA (Messenger RNA)
RT-PCR	Reverse Transcription Polymerase Chain Reaction
SARS-CoV-2	Severe Acute Respiratory Syndrome Coronavirus 2
SESA	Secretaria de Estado da Saúde do Espírito Santo, Vitória, ES, Brazil
SIM	Mortality Information System (Sistema de Informações sobre Mortalidade)
TNF-α	Tumor Necrosis Factor Alpha
Tfh	Follicular Helper T-cells
WHO	World Health Organization
